# Long non-coding RNA MCM3AP-AS1 protects chondrocytes ATDC5 and CHON-001 from IL-1β-induced inflammation via regulating miR-138-5p/SIRT1

**DOI:** 10.1080/21655979.2021.1905247

**Published:** 2021-05-04

**Authors:** Jianming Shi, Fuyang Cao, Yingjian Chang, Chaofei Xin, Xu Jiang, Jianzhong Xu, Shitao Lu

**Affiliations:** Department of Orthopedic Surgery, The First Affiliated Hospital of Zhengzhou University, Zhengzhou, Henan, China

**Keywords:** MCM3AP-as1, miR-138-5p, sirt1, osteoarthritis

## Abstract

Osteoarthritis (OA) is a chronic inflammatory joint disease. Increased apoptosis of chondrocytes contributes to cartilage degradation in OA pathogenesis. The function of lncRNA MCM3AP-AS1 in regulating the viability of chondrocytes still awaits further elaboration. In this work, MCM3AP-AS1, miR-138-5p and SIRT1 mRNA expression levels in OA and normal cartilage tissues were detected by qRT-PCR. Besides, chondrocyte cell lines, CHON-001 and ATDC5 induced by interleukin-1β (IL-1β) were used to initiate the inflammatory response environment of OA. CCK-8 assay was used to examine the cell multiplication; meanwhile, transwell assay was utilized to detect migration. Western blot was adopted to determine SIRT1 expression in chondrocyte. Enzyme-linked immunosorbent assay (ELISA) was performed to evaluate inflammatory factor levels. In addition, the binding sites between MCM3AP-AS1 and miR-138-5p, miR-138-5p and 3ʹUTR of SIRT1 were validated by dual-luciferase reporter assay, RIP assay or RNA pull-down assay. It was found that MCM3AP-AS1 was declined in OA cartilage tissues, positively interrelated with SIRT1 expression while negatively correlated with miR-138-5p. MCM3AP-AS1 up-regulation enhanced the viability and migration of CHON-001 and ATDC5 cells while restraining the apoptosis and inflammatory response. Additionally, miR-138-5p overexpression counteracted the effects on chondrocytes caused by MCM3AP-AS1 overexpression. MCM3AP-AS1 could adsorb miR-138-5p, and SIRT1 was verified as a target of miR-138-5p, and SIRT1 could be up-regulated by overexpression of MCM3AP-AS1 indirectly. In conclusion, MCM3AP-AS1 has the potential to be the ‘ceRNA’ to regulate miR-138-5p and SIRT1 in chondrocytes, and to participate in the pathogenesis of OA.

## 1 Introduction

Osteoarthritis (OA), known as a joint degenerative disease, is featured with inflammatory response, reduction of articular chondrocytes and loss of cartilage matrix [1]. According to statistics, about 60% of the people over 65 years old suffer from OA. Pain, stiffness, swelling and loss of function due to OA can cause severe inconvenience to patients with OA. At present, there are limited methods to treat OA. Drug treatment can only relieve the patients’ pain while joint replacement is expensive [2]. Therefore, it is prominent to clarify the molecular mechanism of OA pathogenesis to delve into new therapy targets for OA. Chondrocytes are necessary for endochondral ossification. Increased chondrocyte apoptosis and excessive inflammatory response contribute to cartilage degradation [3]. Interleukin-1β (IL-1β) is a part of the pathogenesis of OA as an important inflammatory cytokine, and IL-1β exposure can induce chondrocyte inflammation injury *in vitro* [4].

Long non-coding RNA (lncRNA), incapable of encoding proteins, is implicated in many biological processes, like cell proliferation and differentiation, apoptosis, etc. [4,5]. RNA sequencing technology has found that several lncRNAs are differentially expressed in OA development, suggesting that lncRNAs disorders may be vital in the progression of OA [6,7]. LncRNA MCM3AP antisense RNA 1 (MCM3AP-AS1) can expedite the multiplication and invasion of hepatocellular carcinoma cells [8] and papillary thyroid carcinoma cells [9]. Instead, how MCM3AP-AS1 functions in OA is indeterminate.

MicroRNA (miRNA), a class of non-protein coding RNA with length of 18–22*nt*, is closely associated with cell proliferation, differentiation and apoptosis [10]. MiR-138-5p can restrain the multiplication and metastasis of cancer cells in colon cancer [11], lung adenocarcinoma [12] and breast cancer [13]. Additionally, miR-138-5p inhibition can strengthen the growth and migration of chondrocytes, and inhibit the apoptosis and inflammatory response of chondrocytes [14]. A growing number of studies suggest lncRNAs can be the competitive endogenous RNAs (ceRNAs) to modulate miRNAs. Reportedly, MCM3AP-AS1 can promote the multiplication and migration of cancer cells via adsorbing miR-138-5p in pancreatic cancer [15]. However, the interrelations between MCM3AP-AS1 and miR-138-5p in OA remain unknown.

Silent information regulator-1 (SIRT1) is a NAD+-dependent histone deacetylase. SIRT1 is involved in human diseases such as neurodegenerative diseases, diabetes, tumors and multiple inflammatory diseases [16]. Moreover, SIRT1 is in high expression in human articular cartilage tissues, and that the SIRT1 expression in chondrocytes of patients with OA is reduced; SIRT1 can promote chondrocyte survival [17]. SIRT1 can also increase the expression of Bcl-2 by down-regulating the phosphorylation of p38, JNK and ERK, and decreasing Bax, MMP1 and MMP13 expression levels, thus repressing the apoptosis of chondrocytes and the degradation of extracellular matrix of cartilage [18].

The study was designed to investigate the expression characteristics of MCM3AP-AS1, and how MCM3AP-AS1 works in OA. We hypothesized that MCM3AP-1AS1 was down-regulated in the cartilage tissues of OA patients and MCM3AP-AS1 was a ceRNA, which adsorbed miR-138-5p to modulate SIRT1, thereby enhancing chondrocyte viability and restraining inflammatory response.

## 2 Methods and materials

### 2.1 Ethical approval

All experiments in the present work were endorsed by the Ethics Committee of the First Affiliated Hospital of Zhengzhou University (201,701,006), and conducted in compliance with the Declaration of Helsinki. All individuals signed informed consent prior to the collection of tissue samples.

### 2.2 Clinical samples

From January 2017 to March 2019, a total of 30 knee OA patients receiving total knee replacements were collected. Inclusion criteria: patients with OA diagnosed with clinical symptom or image finding [19]. Exclusion criteria were: complicated with rheumatoid arthritis or other autoimmune disorders; contraindications to joint replacement surgery. Kellgren and Lawrence (K-L) grade was scored with radiographic assessments, with 0 representing no abnormalities and 4 representing the most severe lesion [20]. Patient’s age and gender were also recorded. Thirty normal knee cartilages donated after trauma or death were collected as the controls. All samples were stored in liquid nitrogen until RNA extraction.

### 2.3 Cell culture

Human chondrocyte cell line CHON-001 (ATCC® CRL-2846) was purchased from the American Tissue Culture Collection (ATCC, Manassas, VA, USA), and the mouse chondrocyte cell line ATDC5 (BNCC339822) was available from the BeNa Culture Collection (BNCC, Beijing, China). The cells were cultured in Dulbecco’s Modified Eagle Medium (DMEM, Gibco, Grand Island, New York, USA) with 10% fetal bovine serum (FBS, Gibco) and 100 U/mL penicillin and 100 μg/mL streptomycin (Hyclone, Logan, UT, USA) at 37°C with 5% CO_2_. IL-1β (Sigma-Aldrich, Shanghaim China) was subsequently diluted to 1, 10 or 100 ng/mL to treat CHON-001 and ATDC5 cells to stimulate the inflammatory response.

### 2.4 Quantitative real-time polymerase chain reaction (qRT-PCR)

RNA was accordingly extracted from cartilage tissues and cells by TRIzol reagent (Invitrogen, Carlsbad, CA, USA). Notably, reverse transcription of RNA was operated by Primescript ™ reverse transcription kit (TaKaRa, Shiga, Japan) to obtain cDNA, and the DNA amplification was performed on ABI 7500 Real-Time PCR system (Applied Biosystems, Carlsbad, CA, USA) with SYBR Green Master Mix kit (Takara, Otsu, Japan). U6 and GAPDH were employed to normalize gene expression levels and the results were analyzed with 2^−ΔΔCt^ method. Notably, primer sequences are detailed in Table 1.

**Table 1.** The primer sequences used in the present work

Name	Primer sequences
MCM3AP-AS1	Forward: 5ʹ-GCTGCTAATGGCAACACTGA-3’
	Reverse: 5ʹ-AGGTGCTGTCTGGTGGAGAT-3’
MiR-138-5p	5ʹ-AGCTGGTGTTGTGAATCAGG-3ʹ
SIRT1	Forward: 5ʹ-GTATTTATGCTCGCCTTGCTG-3’
	Reverse: 5ʹ-TGACAGAGAGATGGCTGGAA-3’
U6	Forward: 5ʹ -CTCGCTTCGGCAGCACA-3’
	Reverse: 5ʹ -AACGCTTCACGAATTTGCGT-3’
GAPDH	Forward: 5′-ACGGATTTGGTCGTATTGGG-3 ′
	Reverse: 5′-CGCTCCTGGAAGATGGTGAT-3 ′

### 2.5 Cell transfection

MCM3AP-AS1 overexpression plasmid and empty plasmid were from GenePharma (Shanghai, China). Besides, RiboBio (Guangzhou, China) was the provider of miR-138-5p mimics, miR control. Subsequently, the plasmids and miRNAs were, respectively, transfected into chondrocytes with Lipofectamine® 3000 (Invitrogen, Carlsbad, CA, USA) according to the manufacturer’s instruction, and the transfection efficiency was analyzed 48 h after the transfection.

### 2.6 Cell counting kit-8 (CCK-8) assay

CCK-8 kit (Dojindo Laboratories, Kumamoto, Japan) was adopted to detect the multiplication of CHON-001 and ATDC5 cells. These cells were, respectively, transferred into a 96-well plate (cell density: 1 × 10^5^ cells/well). To probe the viability of the cells, 10 μL of CCK-8 reagent was loaded into each well, and cells were accordingly incubated at 37°C with 5% CO_2_ for 2 h. Following that, a microplate reader (Model 550, Bio-Rad Laboratories, Inc., Hercules, CA, USA) was adopted to detect the optical density (OD) value at 450 nm.

### 2.7 Western blot

The total protein extracted by RIPA buffer was subsequently quantified with BCA protein detection kit (Beyotime, Shanghai, China). The protein samples in different groups, were, respectively, mixed with the loading buffer, and then immediately heated in boiling water for 10 min. Then, the proteins were dissolved by SDS-PAGE and accordingly transferred onto the PVDF membrane (Beyotime, Shanghai, China), which was then blocked in 5% skimmed milk at ambient temperature for 2 h. Following that, the primary antibodies against SIRT1 and GAPDH (Proteintech, Wuhan, China) were used to incubate the PVDF membranes at 4°C for 12 h. Rinsed for three times with TBST buffer, the membranes were immediately incubated with secondary antibody (Proteintech, Wuhan, China) for 1 h at room temperature. The membranes were then rinsed three times with TBST buffer again, and an enhanced chemiluminescence substrate kit (Amersham Pharmacia Biotech, Little Chalfont, UK) was added onto the membranes, and the protein bands were developed.

### 2.8 Transwell assay

CHON-001 and ATDC5 cells were suspended with DMEM containing 1% FBS, then were inoculated in the upper compartment of the Transwell system (Costar, Cambridge, MA, USA) (1 × 10^5^ cells/well), and DMEM with 10% FBS was accordingly loaded into the lower compartment. The cells were then cultured for 48 h. Next, the chondrocytes remaining on the upper surface of the membrane were wiped off, and the cells on the lower surface of the membrane were immediately fixed in paraformaldehyde and subsequently stained with crystal violet solution. Finally, the chondrocytes in each group were observed under a microscope (Olympus, Tokyo, Japan), and the number of the cells is recorded.

### 2.9 Flow cytometry

Cell apoptosis rate in each group was respectively measured using an Annexin V-FITC apoptosis detection kit (Beyotime, Shanghai, China) and propidium iodide (PI). Specifically, ATDC5 or CHON-001 cells (1 × 10^5^ cells/group) were trypsinized and rinsed with phosphate buffer solution; then, they were collected after centrifugation. Following that, the cells in each group were immediately resuspended with 500 µL of binding buffer (Beyotime, Shanghai, China). Following that, 10 μL of Annexin V-FITC staining solution was added into the binding buffer, and the cells were incubated in darkness for 15 min, and then 5 μL of propidium iodide (PI) staining solution were added, and the cells were incubated for another 5 min. The apoptosis of the cells was then examined by a flow cytometer (FACS CaliburTM, BD, Franklin Lakes, NJ, USA).

### 2.10 Enzyme-linked immunosorbent assay (ELISA)

IL-6, IL-8, and TNF-α contents in the supernatant of ATDC5 or CHON-001 cells were measured by the corresponding ELISA kit (Ebioscience, San Diego, CA, USA), according to instructions provided by the manufacturer.

### 2.11 Bioinformatics analysis

StarBase online database (starbase.sysu.edu.cn/) was adopted to predict the targeting relationships among MCM3AP-AS1, miR-138-5p and SIRT1.

### 2.12 Dual-luciferase reporter assay

The binding sites between MCM3AP-AS1/miR-138-5p and miR-138-5p/SIRT1 were confirmed by a dual-luciferase reporter gene assay. Specifically, the reporter vectors and miRNAs were co-transfected in to HEK293T cells, and then the luciferase activity in each group was, respectively, examined by Dual Luciferase Reporter Gene Assay Kit (Promega, Madison, WI, USA). To analyze the results, firefly luciferase activity was normalized to Renilla luciferase activity.

### 2.13 RNA-binding protein immunoprecipitation (RIP) assay

RIP assay was performed with Magna RIP™ RNA-Binding Protein Immunoprecipitation Kit (Millipore, Billerica, MA, USA). In short, the cells in each group were lysed in RIP lysis buffer, and then incubated with the magnetic beads conjugated with anti-Ago2 or anti-IgG antibodies. After the immunoprecipitate was isolated, Proteinase K buffer was loaded to the samples to remove the proteins. Ultimately, the enrichment of miR-138-5p and SIRT1 mRNA in the immunoprecipitate was examined by qRT-PCR.

### 2.14 RNA pull-down assay

In brief, 0.5 ml buffer (25 mM Tris-HCl, 0.05% NP-40, 70 mM KCl, 2.5 mM EDTA, 80 U/ml RNase inhibitor and 1× protease inhibitor) were used to incubate CHON-001 and ATDC5 on ice. After centrifugation for 20 min, the supernatant was immediately collected. Following that, biotin-coupled miR-138-5p (bio-miR-138-5p) or biotin-coupled control miRNA (bio-NC) (RiboBio, Guangzhou, China) was accordingly mixed with the supernatant and incubated for 2 h. The streptavidin magnetic beads were then loaded. 4 h later, the streptavidin magnetic beads were accordingly isolated, and the enrichment of SIRT1 mRNA enrichment was quantified via qRT-PCR in the isolated RNA.

### 2.15 Statistical analysis

The aforementioned experiments were conducted for at least three times. SPSS software (version 22.0, IBM Corp., Armonk, NY, USA) was adopted for the data analysis. The data were in the form of ‘mean ± standard deviation’. Notably, the data in the two groups were subsequently compared by Student’s *t*-test. Additionally, the data in three or more groups were compared by one-way ANOVA and comparisons between the two groups were then performed using LSD post-hoc test. Correlations among the gene expression levels were analyzed using Pearson’s correlation analysis. The relationship between the MCM3AP-AS1 expression and OA patients’ characteristics was analyzed using chi-square test. Notably, *P* < 0.05 is statistically meaningful.

## 3. Results

Our study was aimed to investigate how MCM3AP-AS1 worked in OA pathogenesis. With IL-1β to stimulate CHON-001 and ATDC5 cells, we hypothesized that MCM3AP-AS1 could adsorb miR-138-5p to modulate SIRT1, thereby enhancing chondrocyte viability and restraining inflammatory response.

### 3.1 IL-1β induces inflammatory injury in chondrocytes in vitro

**Figure 1.**
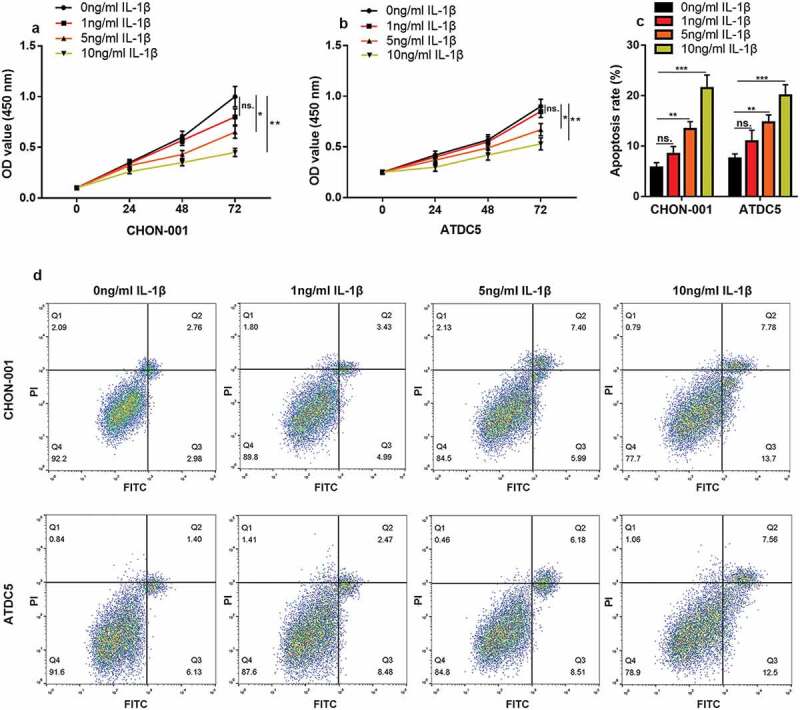
***IL-1β induces inflammatory injury to chondrocytes in vitro.*** (a, b) CHON-001 and ATDC5 cells were treated with IL-1β (0 ng/mL, 1 ng/mL, 5 ng/mL, 10 ng/mL), and the viability of the cells was measured by CCK-8 assay. (c, d) Flow cytometry was employed to measure the apoptosis rate of CHON-001 and ATDC5 cells. ns.: not statistically significant

First, CHON-001 and ATDC5 cells were treated with IL-1β (0 ng/mL, 1 ng/mL, 5 ng/mL, or 10 ng/mL) to construct an *in vitro* OA model. CCK-8 assay highlighted that the cell viability in 5 ng/mL and 10 ng/mL groups was considerably lower than that of the control (Figure 1a & B). Flow cytometry depicted that the apoptosis rate of chondrocytes was greatly elevated in the 5 ng/mL and 10 ng/mL groups (Figure 1c). In the following experiments, 10 ng/mL IL-1β was selected as the stimulating condition.

*P < 0.05, **P < 0.01, and ***P < 0.001 compared with the control.

***3.2 MCM3AP-AS1 expression level significantly declined in OA cartilage tissues and IL-1β-treated chondrocytes***

**Figure 2.**
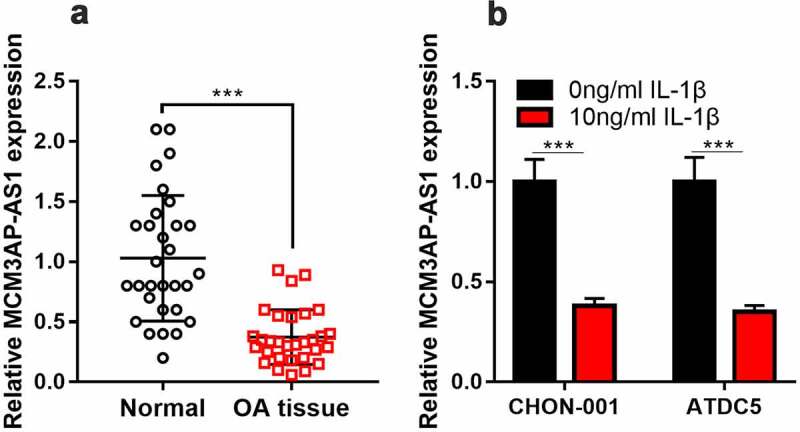
***MCM3AP-AS1 expression in OA.*** (a, b) qRT-PCR showed that MCM3AP-AS1 was significantly down-regulated in the cartilage tissues of OA patients and OA models *in vitro.*

To figure out whether MCM3AP-AS1 expression was changed in OA tissues and OA cell models, qRT-PCR was performed. It is shown that MCM3AP-AS1 has markedly decreased in OA articular cartilage tissues, as against the normal cartilage tissues (Figure 2a). Besides, compared with untreated cells, MCM3AP-AS1 was remarkably down-regulated in 10 ng/mL IL-1β-treated CHON-001 and ATDC5 cells (Figure 2b). Subsequently, according to the expression level of MCM3AP-AS1, the patients were on average grouped into high- and low-expression groups. Chi-square test showed that lower expression of MCM3AP-AS1 in the articular cartilage tissues was correlated with higher K-L grade (Table 2).

**Table 2.** Relationship between MCM3AP-AS1 expression and clinicopathological characteristics of knee OA

Characteristic	Cases	MCM3AP-AS1		P-value
		Low (n = 15)	High (n = 15)	
Age (yr)				
≥ 60	17	7	10	0.269
< 60	13	8	5	
Gender				
Male	11	6	5	0.704
Female	19	9	10	
K-L category				
III-Ⅳ	14	10	4	0.028*
I–II	16	5	11

***P < 0.001 compared with the control.

***3.3 MCM3AP-AS1 overexpression promotes chondrocyte viability and migration, inhibits apoptosis and inflammatory response in OA model***

**Figure 3.**
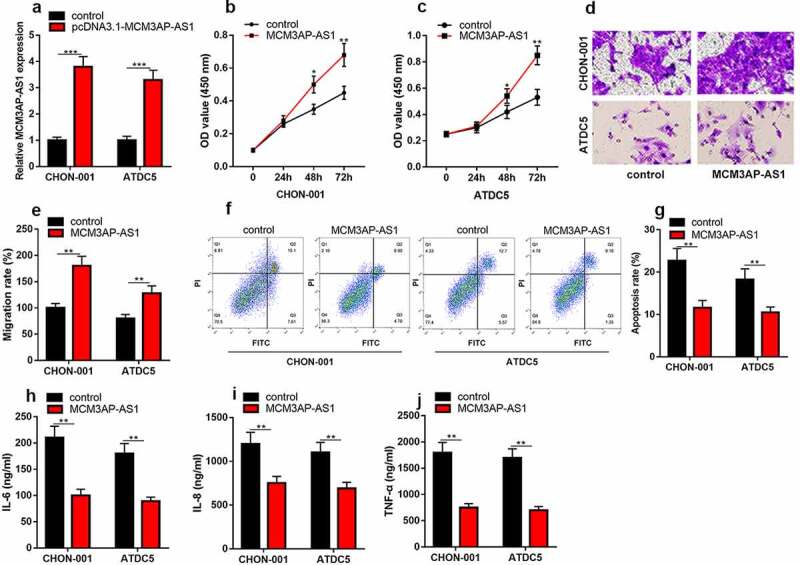
***Overexpression of MCM3AP-AS1 promotes the viability of chondrocytes, inhibits apoptosis and inflammatory response in OA models in vitro.*** (a) qRT-PCR showed that the MCM3AP-AS1 overexpression model was successfully constructed. (b, c) CCK-8 assay showed that overexpression of MCM3AP-AS1 significantly promoted the viability of chondrocytes. (d, e) Transwell assay showed that overexpression of MCM3AP-AS1 significantly promoted the migration of chondrocytes. (f, g) Flow cytometry assay showed that overexpression of MCM3AP-AS1 significantly inhibited apoptosis of chondrocytes. (H, I, J) ELISA assay showed that overexpression of MCM3AP-AS1 significantly inhibited the levels of IL-6, IL-8 and TNF-α in OA models *in vitro.*

To delve into how MCM3AP-AS1 functioned in OA progression, we successfully constructed the MCM3AP-AS1 overexpression models in IL-1β-stimulated CHON-001 and ATDC5 chondrocyte cell lines (Figure 3a). CCK-8 assay uncovered that compared with the control group, MCM3AP-AS1 up-regulation dramatically promoted the viability (Figure 3b & C). Transwell assay uncovered that MCM3AP-AS1 overexpression could promote the migration of chondrocytes (Figure 3d & E). Flow cytometry proved that the apoptosis in the MCM3AP-AS1 overexpression group was greatly suppressed than that in the control group (figure 3f & G). As against the control group, the contents of IL-6, IL-8 and TNF-α in MCM3AP-AS1 overexpression group were dramatically declined (Figure 3h-j).

*P < 0.05, **P < 0.01, and ***P < 0.001 compared with the control.

### 3.4 MiR-138-5p is a target of MCM3AP-AS1

**Figure 4.**
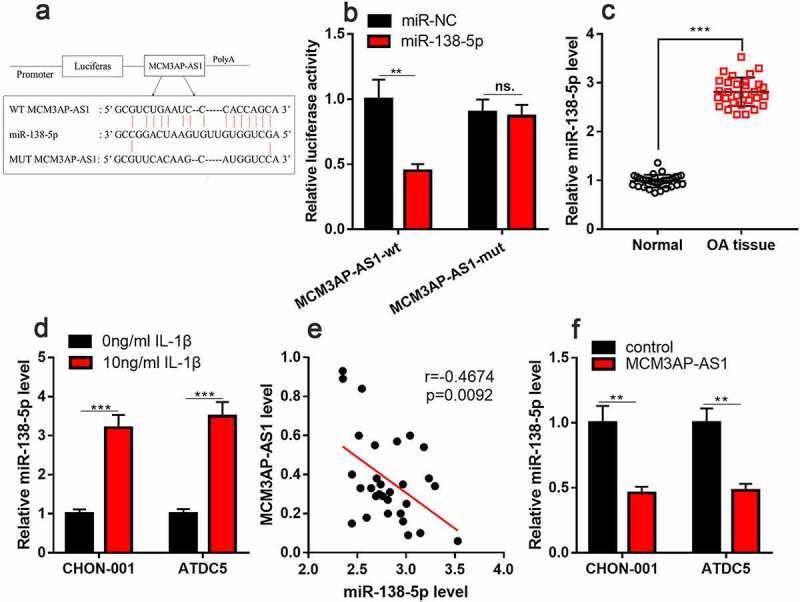
***MiR-138-5p is the target of MCM3AP-AS1.*** (a) StarBase database showed that miR-138-5p may be a potential target for MCM3AP-AS1. (b) Dual-luciferase reporter gene assay depicted that miR-138-5p mimics can significantly reduce the luciferase activity of wild-type MCM3AP-AS1 reporter, and has no effect on that of mutant MCM3AP-AS1 reporter. (c) qRT-PCR suggested that miR-138-5p was up-regulated in the cartilage tissues of OA patients. (d) qRT-PCR indicated that IL-1β treatment induced miR-138-5p expression in chondrocytes. (e) qRT-PCR data indicated that MCM3AP-AS1 and miR-138-5p expression levels were negatively correlated in the cartilage tissues of OA patients. (f) MiR-138-5p expression in the chondrocytes of MCM3AP-AS1 overexpression group were significantly reduced. ns.: not statistically significant

StarBase database predicted that miR-138-5p could probably be a target of MCM3AP-AS1 (Figure 4a). Besides, dual-luciferase reporter assay found that miR-138-5p mimics can considerably impede the luciferase activity of wild-type MCM3AP-AS1 reporter and have no obvious impact on that of mutant MCM3AP-AS1 reporter (Figure 4b). qRT-PCR implied that miR-138-5p was elevated in the cartilage tissues of OA patients and chondrocytes treated with IL-1β (Figure 4c & D). Additionally, MCM3AP-AS1 and miR-138-5p expression levels were negatively correlated in the cartilage tissues of OA patients (Figure 4e). As against the control, miR-138-5p expression was inhibited in MCM3AP-AS1 overexpression group (figure 4f). These data highlighted that MCM3AP-AS1 could probably adsorb miR-138-5p to repress its expression in chondrocytes.

**P < 0.01, and ***P < 0.001 compared with the control.

### 3.5 MiR-138-5p counteracts the effects of MCM3AP-AS1 in chondrocytes

**Figure 5.**
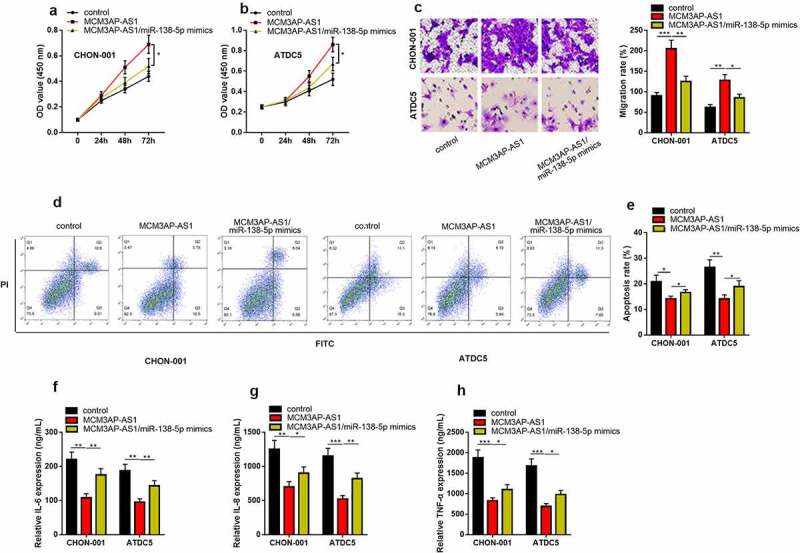
***MiR-138-5P reverses the role of MCM3AP-AS1 in OA chondrocytes.*** (a, b) CCK-8 assay results showed that the effect of MCM3AP-AS1 overexpression on cell proliferation could be offset by miR-138-5 mimics. (c) Transwell assay indicated that the effect of MCM3AP-AS1 overexpression on the migration of chondrocytes could be counteracted by miR-138-5p (d) Flow cytometry showed that the decrease in apoptosis rate induced by overexpression of MCM3AP-AS1 can be offset by miR-138-5 mimics. (E, F, G, H) ELISA results showed that the contents of inflammatory cytokines after cotransfection of miR-145-5p was significantly higher than those in MCM3AP-AS1 overexpression group

We further analyzed whether MCM3AP-AS1 could be a vital modulator in OA through regulating miR-138-5p. CCK-8 and Transwell experiments highlighted that the promoting impact of MCM3AP-AS1 overexpression on cell viability and migration could be counteracted by miR-138-5 mimics (Figure 5a&B&C). Flow cytometry implied that overexpression of MCM3AP-AS1 repressed the apoptosis of chondrocytes, which would be counteracted by the co-transfection of miR-138-5 mimics (Figure 5d & E). ELISA manifested that, the contents of inflammatory factor IL-6, IL-8, TNF-α were higher than those in MCM3AP-AS1 overexpression group after the co-transfection of miR-145-5p mimics, suggesting the inflammatory response was aggravated by miR-138-5p (figure 5f-h).

*P < 0.05, **P < 0.01, and ***P < 0.001.

### 3.6 MCM3AP-AS1 regulated SIRT1 expression through miR-138-5p

**Figure 6.**
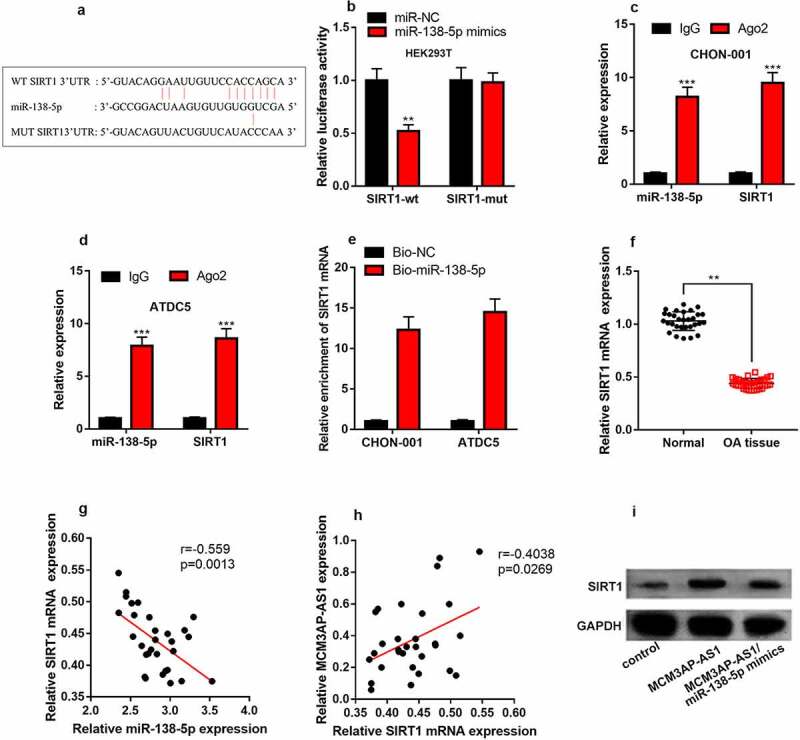
***MCM3AP-AS1 regulates SIRT1 expression through miR-138-5p.*** (a) SIRT1 contained a binding sequence for miR-138-5p. (b) Dual-luciferase reporter gene assay confirmed that miR-138-5p mimics reduced the luciferase activity of the wild-type SIRT1 reporter, but had no effect on the luciferase activity of the mutant SIRT1 reporter. (c, d) RIP assay showed that miR-138-5p could directly bind with SIRT1 mRNA. (e) The RNA pull-down assay was performed to validate the interaction between miR-138-5p and SIRT1 mRNA. (f) qRT-PCR showed that SIRT1 mRNA was significantly down-regulated in OA cartilage tissues. (g) There was a negative correlation between miR-138-5p and SIRT1 mRNA expression levels in OA cartilage tissues. (h) There was a positive correlation between MCM3AP-AS1 and SIRT1 mRNA expression levels in OA cartilage tissues. (i) Overexpression of MCM3AP-AS1 could significantly increase SIRT1 expression. After co-transfection of MCM3AP-AS1/miR-138-5p, SIRT1 expression was significantly reduced. ns.: not statistically significant

StarBase database also predicted that SIRT1 3ʹUTR contained a binding sequence for miR-138-5p (Figure 6a). Dual-luciferase reporter gene assay depicted that miR-138-5p mimics restrained the luciferase activity of wild-type SIRT1 reporter, but had no effect on that of mutant SIRT1 reporter (Figure 6b). RIP assay highlighted that miR-138-5p could interact with SIRT1 mRNA in both cell lines (Figure 6c&D). RNA pull-down assay uncovered that SIRT1 mRNA was specifically enriched by biotin-coupled miR-138-5p in CHON-001 and ATDC5 cells (Figure 6e). qRT-PCR indicated that compared with normal cartilage tissues, the expression level of SIRT1 mRNA was reduced in OA cartilage tissues (figure 6f), and miR-138-5p and SIRT1 mRNA expression levels were negatively correlated in the tissues (Figure 6g), and MCM3AP-AS1 expression level was positively correlated with SIRT1 mRNA expression level (Figure 6h). Western blot depicted that in CHON-001 cells, as against the control, MCM3AP-AS1 overexpression remarkably increased SIRT1 expression, but SIRT1 expression was significantly reduced after co-transfection of miR-138-5p mimics (Figure 6i), and these data suggested that MCM3AP-AS1/miR-138-5p axis regulated SIRT1 expression in chondrocytes.

*P < 0.05, **P < 0.01, and ***P < 0.001.

## 4. Discussion

OA is a heterogeneous disease featuring with articular cartilage degeneration, which mainly affects hip joint and knee joint [1]. Chondrocyte apoptosis is related to cartilage destruction in patients with OA [21]. Recently, increasing studies have affirmed that many lncRNAs features prominently in the pathogenesis of OA. For instance, lncRNA DNM3OS is lowly expressed in OA cartilages, and DNM3OS up-regulation can promote viability, migration and inhibit apoptosis of chondrocytes [22]; lncRNA SNHG1 can reduce IL-1β-induced inflammatory response in chondrocytes by restraining miR-16-5p-mediated p38 MAPK and NF-kB signaling pathways [23]. As reported, MCM3AP‐AS1 is pivotal in many human diseases. For instance, MCM3AP-AS1 can enhance cell growth and metastasis in colorectal cancer via modulating miR-193a-5p/SENP1 [24]. MCM3AP-AS1 expedites cell multiplication and invasion through modulating miR-543-3p/SLC39A10/PTEN axis in prostate cancer [25]. In addition, MCM3AP-AS1 modulates miR-142-3p/HMGB1 axis to participate in lipopolysaccharide-inducedchondrocyte apoptosis [26]. Here we proved that MCM3AP-AS1 expression level was greatly reduced in OA cartilages and chondrocytes induced by IL-1β, and overexpression of MCM3AP-AS1 improved the proliferation and migration of chondrocytes, and impeded the apoptosis and inflammation. MCM3AP-AS1 low-expression was also proved to be associated with higher K-L grade. The aforementioned findings uncovered that MCM3AP-AS1 was a protective factor for chondrocytes and cartilage, and its down-regulation contributed to OA development.

Mounting studies have depicted that miRNA is vital in the OA progression via modulating the apoptosis and differentiation of chondrocytes, such as miRNA-103 [27], miRNA-455-3p [28] and miR-33b-3p [29]. Reportedly, miR-183-5p is significantly up-regulated in OA tissue [30], which can promote the apoptosis of chondrocytes induced by IL-1β [14]. LncRNAs can function as ceRNAs to adsorb miRNAs like molecular sponges, and exert biological functions on OA. For example, lncRNA SNHG5 can sponge miR-26a to accelerate the multiplication of chondrocytes [31]. LncRNA DANCR regulates the progression of OA through modulating miR-577/Sphk2 axis [32]. Herein, we found that miR-183-5p was a target of MCM3AP-AS1. With MCM3AP-AS1 up-regulation, miR-183-5p expression in chondrocytes was decreased, and the impacts of MCM3AP-AS1 on chondrocytes could be partially counteracted by miR-183-5p. Therefore, we conclude that MCM3AP-AS1 can adsorb miR-183-5p to regulate the biological behaviors of chondrocytes. The regulatory relationship between MCM3AP-AS1 and miR-183-5p has been reported in pancreatic cancer [15], which is consistent with our findings.

**Figure 7.**
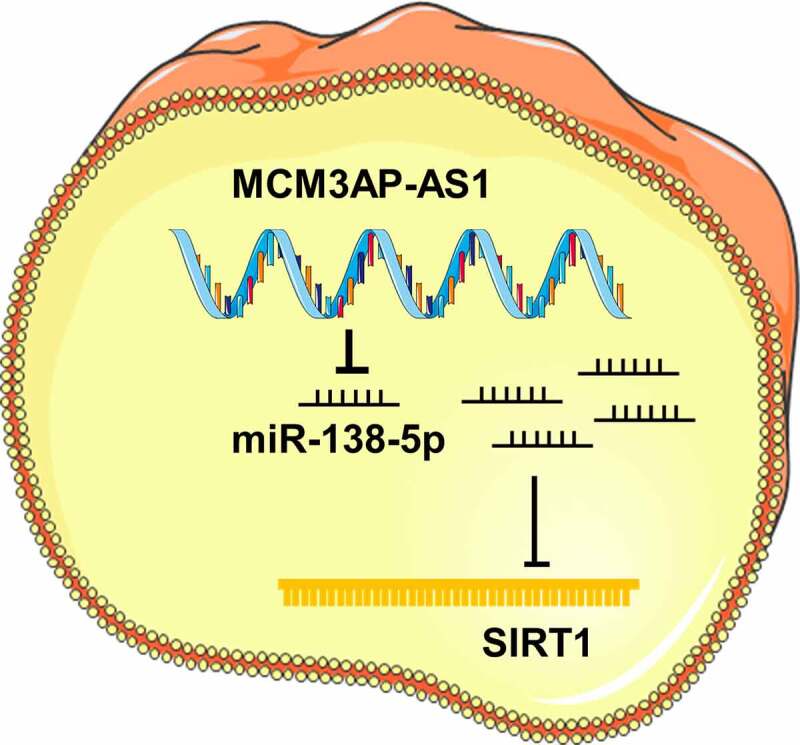
Graphic abstract: MCM3AP-AS1 functioned through the miR-138-5p/SIRT1 axis in chondrocytes to regulate the progression of OA, showing protective effects

SIRT1 participates in regulating multiple important biological processes, including stress response, DNA repair and inflammation. SIRT1 is a vital regulator in age-related diseases, such as type 2 diabetes and Alzheimer’s disease [33]. It is reported that SIRT1 is pivotal in the progression of OA. High expression of SIRT1 in chondrocytes can activate IGFR and downstream PI3K, PDK1, mTOR and Akt, thereby phosphorylating MDM2, inhibiting p53, and preventing chondrocyte apoptosis [34]. Besides, SIRT1 down-regulation can promote human chondrocyte apoptosis by regulating mitochondria apoptosis pathway [35]. It is well known that miRNAs can repress translation via binding to the 3′ untranslated region (3′UTR) of the target mRNA [36]. Our data showed that compared with normal tissues, SIRT1 mRNA was greatly inhibited in OA cartilage tissues, and miR-138-5p expression level was negatively correlated with SIRT1 mRNA expression level. Through bioinformatics methods, dual-luciferase reporter gene assay, RIP assay and RNA pull-down assay, we proved that miR-138-5p can directly bind to SIRT1 3′UTR. Additionally, miR-138-5p mimics reversed the up-regulation of SIRT1 induced by MCM3AP-AS1 overexpression. The above evidence suggests that MCM3AP-AS1 functioned through the miR-138-5p/SIRT1 axis in chondrocytes to regulate the progression of OA (Figure 7).

## 5. Conclusion

We demonstrate that MCM3AP-AS1 expression is dramatically reduced in the cartilage tissues of OA patients. It is also revealed that MCM3AP-AS1 improves the viability of chondrocytes via modulating the miR-138-5p/SIRT1 axis and constrains apoptosis and inflammatory responses. This study partly clarifies the molecular mechanism of OA pathogenesis, and provides potential targets for OA treatment. In the following work, animal models are needed to further verify our demonstrations in the present work.

## Highlights

1. MCM3AP-AS1 was significantly downregulated in OA cartilage tissues and IL-1β-treated chondrocytes;
2. Overexpression of MCM3AP-AS1 promoted chondrocyte viability and migration, inhibited apoptosis and inflammatory response in OA model;
3. MCM3AP-AS1 up-regulated SIRT1 expression through repressing miR-138-5p in chondrocytes.
